# General Public’s knowledge, awareness, and perception of Cardiometabolic diseases: data from a Singapore study population

**DOI:** 10.3389/fmed.2023.1193829

**Published:** 2023-04-24

**Authors:** Vickram Vijay Anand, Rachel Sze Jen Goh, Benjamin Nah, Sky Wei Chee Koh, Jieyu Lim, Nicholas W. S. Neo, Jocelyn Chew, Yuan Ying Lee, Yip Han Chin, Bryan Chong, Gwyneth Kong, Bryan Tan, Zhiwen Low, Chin Meng Khoo, Lay Hoon Goh, Poay Huan Loh, Ping Chai, Mayank Dalakoti, Mark Chan, Roger Foo, Mark Muthiah, Nicholas W. S. Chew

**Affiliations:** ^1^Lee Kong Chian School of Medicine, Nanyang Technological University, Singapore, Singapore; ^2^Yong Loo Lin School of Medicine, National University Singapore, Singapore, Singapore; ^3^Division of Gastroenterology and Hepatology, Department of Medicine, National University Hospital, Singapore, Singapore; ^4^National University Polyclinics, National University Health System, Singapore, Singapore; ^5^Division of Family Medicine, Yong Loo Lin School of Medicine, National University of Singapore, Singapore, Singapore; ^6^Nursing Department, National University Heart Centre, Singapore, Singapore; ^7^Alice Lee Centre for Nursing Studies, Yong Loo Lin School of Medicine, National University Singapore, Singapore, Singapore; ^8^Faculty of Science, Monash University, Melbourne, VIC, Australia; ^9^Department of Endocrinology, National University Hospital, Singapore, Singapore; ^10^Department of Cardiology, National University Heart Centre, National University Health System, Singapore, Singapore; ^11^Division of Cardiology, Department of Medicine, Ng Teng Fong General Hospital, Singapore, Singapore; ^12^National University Centre for Organ Transplantation, National University Health System, Singapore, Singapore

**Keywords:** knowledge, awareness, perception, actions, cardiometabolic disease, risk factors

## Abstract

**Background:**

Health literacy and illness perception play crucial roles in tackling the cardiometabolic disease epidemic. We aim to compare the attitudes, knowledge, self-perceived risks and actions taken, between individuals with and without metabolic risk factors (MFs).

**Methods:**

From 5 June to 5 October 2022, participants of the general public were invited to complete a self-administered questionnaire. MF status was defined as the presence of hypertension, hyperlipidemia, diabetes mellitus and/or current/previous smoking. Participants were assessed based on four categories (knowledge-based, attitude-based, perceived risk, and action-based) of questions pertaining to four cardiometabolic diseases – diabetes mellitus, hypertension, hyperlipidemia, and non-alcoholic fatty liver disease.

**Results:**

A total of 345 participants were enrolled, of whom 34.5% had at least one MF. Compared to those without MFs, participants with MFs had lower knowledge scores, but higher perceived risk scores across all cardiometabolic diseases. The largest knowledge gap pertained to hypertension-related questions. After adjustment, linear regression demonstrated that the presence of MFs (β:2.752, 95%CI: 0.772–4.733, *p* = 0.007) and higher knowledge scores (β:0.418, 95%CI: 0.236–0.600, *p* < 0.001) were associated with higher perceived risk. Despite increased perceived risk in those with MFs, this translated to only few increased self-reported preventive actions, when compared to those without MFs, namely the reduction in red meat/processed food consumption (*p* = 0.045) and increase in fruits/vegetables consumption (*p* = 0.009).

**Conclusion:**

This study identified a vulnerable subpopulation living with MFs, with high perceived risks, and discordant levels of knowledge and preventive actions taken. Nationwide efforts should be channeled into addressing the knowledge-to-action gap.

## 1. Introduction

The rising cardiometabolic disease epidemic is a global health concern, with worrisome trends of disease burden and morbidity over the past decades (1–3). In Asia, nearly a fifth of the adult population is affected by cardiometabolic diseases (4). Based on the National Population Health Survey 2021 (5), the prevalence of hypertension and hyperlipidemia in Singapore was 15.7 and 13.9% respectively, while the prevalence of diabetes mellitus and was 6.9%. Cardiometabolic diseases exist in tandem with one another, often sharing similarities in underlying pathomechanisms including insulin resistance and metabolic dysregulation. In the later stages, they can manifest as clinically identifiable pre-disease states of metabolic syndrome and prediabetes, eventually resulting in cardiovascular diseases and diabetes mellitus (6). In contrast to Western populations, there is a higher prevalence of metabolic dysfunction at relatively lower levels of obesity in Asian populations, which makes the identification and management of these silent metabolic disorders even more challenging (7–10). Increasingly, the individual’s own illness perception and understanding of their medical condition have been shown to have significant effects on readmission rates and recovery for many diseases (11, 12). This has been attributed to higher medication compliance (13, 14) and adherence to lifestyle changes (15) in individuals with better disease knowledge and positive illness perceptions. With multi-disciplinary approaches to managing cardiometabolic disease often hinging on lifestyle modifications and medication adherence as the cornerstone of primary prevention (16–19), there is an urgent need to understand the public’s knowledge and illness perception that can prove useful for important stakeholders in healthcare policymaking in the concerted efforts to derail the incoming metabolic wave (20–23).

While the current literature has explored the perception of cardiometabolic disease in the Western population (24), there is limited data on the knowledge and perception of cardiometabolic diseases within the Singaporean population (25, 26). Given the unique cultural and socio-economic factors that influence risk perception and actions (27), this study aims to provide insights into the perception of cardiometabolic diseases the Singapore population by exploring the general public’s attitudes and knowledge of the disease, self-perceived risks and actions taken to improve metabolic health, stratified by the presence of metabolic risk factors (MFs). These unique perspectives can help inform policy makers on the vital components of cardiometabolic diseases that needs to be addressed, as well as the identification of vulnerable population subgroups that may benefit from targeted interventions.

## 2. Materials and methods

### 2.1. Study design and population

This cross-sectional study was conducted from 5 June 2022 to 5 October 2022. Participants were recruited through convenience sampling. The online survey was disseminated to personal contacts, *via* social media and through mailing lists, and participants forwarded the survey link to other individuals that met the eligibility criteria. The study was open to all participants aged 21 years and above, residing in Singapore.

### 2.2. Data collection

All participants willing to participate in the study filled in an online self-administered English questionnaire secured by FormSg. Implied consent was indicated through the voluntary completion of the online questionnaire. Baseline characteristics such as age, sex, ethnicity, and anthropometric variables were recorded. Participants were stratified into two groups, participants with MFs and those without. The presence of MFs was defined as the self-reported presence of hypertension, hyperlipidemia, diabetes mellitus and current or ex-smoking.

### 2.3. Instrument

The questionnaire was developed and adapted for the use in our Singaporean cohort, based on previous validated psychometric tools (28–32). It is sectioned into four main categories – knowledge-based, attitude-based, risk perception, and action-based questions. Knowledge-based questions evaluated participants’ understanding of the metabolic diseases including their awareness of the disease, perception of the risk factors, reversibility, and complications of the disease. Attitude-based questions examined the perceived importance of factors including lifestyle modifications and medications in improving the course of the disease. Risk perception questions measured both the cognitive and affective aspect of patients’ perception of susceptibility to developing complications of cardiometabolic diseases. Action-based questions evaluated participants’ willingness in taking action toward mitigating cardiometabolic risks. These questions were raised in the survey in relation to each of the four cardiometabolic diseases that were being studied, namely hypertension, hyperlipidemia, diabetes mellitus and non-alcoholic fatty liver disease (NAFLD). The study was approved by the local institutional review committee in accordance with the revised Declaration of Helsinki (National Healthcare Group Domain Specific Review Board Ref: 2022/00097).

### 2.4. Statistical analysis

The study outcomes included participants’ knowledge and attitude toward cardiometabolic diseases, their perceived risk of cardiometabolic complications, and actions taken to mitigate these risks, based on the individual’s MF status.

Knowledge scores were computed in accordance to the latest guideline recommendations (33, 34), with the objective of gaging participants’ understanding of specific cardiometabolic diseases. The knowledge-based point system was developed with reference to a study (35) by Al-Hanawi et al. For knowledge questions on a Likert scale, scores were assigned based on the following: “Strongly Disagree,” “Disagree” and “Neutral” were assigned 0 points, while “Agree” and “Strongly Agree” were assigned 1 point. For the remaining questions, correct responses were assigned a score of 1, and incorrect or uncertain answers were assigned a score of 0. Perceived risk scores comprising of both the affective and cognitive component were developed to evaluate the extent of the participants’ perceived susceptibility to cardiometabolic diseases and the complications (32). The cognitive aspect was assessed on the following scale (“Very High Risk” = 5 points, “High Risk” = 4 points, “Neutral” = 3 points, “Low Risk” = 2 points, “Very Low Risk” = 1 point). Higher perceived risk scores signified that the individual perceives their own risk of developing an adverse event to be increased. The affect aspect, participants’ level of concern was evaluated on a scale (“Not Worried at All” = 1 point, “A Little Worried” = 2 points, “Somewhat Worried” = 3 points, “Worried” = 4 points and “Very Worried” = 5 points). Based on the cumulated perceived risk score, a multivariable linear regression model was constructed to examine independent predictors of increased perceived risk, which included covariates such as age, sex, ethnicity, marital status, education levels, presence of MFs, and knowledge-based score (36).

The analysis was conducted using IBM SPSS statistics 25 (SPSS Inc., Chicago, IL) and RStudio (Version 4.2.2). Categorical variables were expressed as numbers (percentages) and continuous variables were expressed as mean (standard deviation). Pearson’s chi-square test was used to compare categorical variables, 2-sample T test (or Mann–Whitney U test, where appropriate) for the comparison of continuous and ordinal variables. A *p* <0.05 was deemed statistically significant for this study.

## 3. Results

### 3.1. Baseline characteristics

A total of 345 participants were recruited for this study, of whom 226 (65.5%) were participants without MFs and 119 (34.5%) were participants with MFs. Individuals with MFs tended to be older (55 ± 11 years) and more likely to be male (44.5%), compared to those without MFs (37 ± 12 years, 35.8% male). Ethnicity did not vary significantly between the groups, with the majority being Chinese (89.0%), followed by Malay (5.8%), and Indian (2.6%) within the overall population. Of the study cohort, 7.0% of participants had the diagnosis of diabetes mellitus, 3.8% had prediabetes, 16.8% had hypertension, 26.8% had hyperlipidemia, and 10.5% were current/ex-smokers. The demographics and clinical characteristics of participants are appended in Table 1. All participants completed the surveys without any missing data.

**Table 1:** Baseline demographics and clinical characteristics.

Variable	Overall Cohort (*N* = 345)	Metabolic risk factors (*N* = 119)	No metabolic risk factors (*N* = 226)	*p*-values
Male sex	134 (38.8%)	53 (44.5)	81 (35.8)	0.144
Ethnicity				0.097
Chinese	307 (89.0%)	105 (88.2)	202 (89.4)	
Malay	20 (5.8%)	7 (5.9)	13 (5.8)	
Indian	9 (2.6%)	1 (0.8)	8 (3.5)	
Others	9 (2.6%)	6 (5.0)	3 (1.3)	
Age	43.9 (14.6)	55.7 (11.1)	37.7 (12.3)	**<0.001**
Body Mass Index, kg/m^2^	23.3 (4.2)	24.3 (4.4)	22.7 (4.1)	**0.001**
Education status				**0.002**
Secondary Education	80 (23.2%)	41 (34.5%)	39 (17.3%)	
Tertiary Education	180 (52.2%)	53 (44.5%)	127 (56.2%)	
Post-Graduate	85 (24.6%)	25 (21.0%)	60 (26.5%)	
Employment status				**<0.001**
Student	10 (2.9%)	0 (0.0%)	10 (4.4%)	
Unemployed	18 (5.2%)	10 (8.4%)	8 (3.5%)	
Working (Part-time)	26 (7.5%)	13 (10.9%)	13 (5.8%)	
Working (Full-time)	261 (75.7%)	72 (60.5%)	189 (83.6%)	
Retired	30 (8.7%)	24 (20.2%)	6 (2.7%)	
Income				**0.049**
Less than SGD$1,000	40 (11.6%)	17 (14.3%)	23 (10.2%)	
SGD$1,001–2,500	23 (6.7%)	12 (10.1%)	11 (4.9%)	
SGD$2,501–5,000	117 (33.9%)	34 (28.6%)	83 (36.7%)	
SGD$5,001–7,500	73 (21.2%)	18 (15.1%)	55 (24.3%)	
SGD$7,501–10,000	39 (11.3%)	15 (12.6%)	24 (10.6%)	
More than SGD$10,000	53 (15.4%)	15 (12.6%)	30 (13.3%)	
Diabetes Mellitus	24 (7.0%)	24 (20.2%)	-	**<0.001**
Pre-diabetes	13 (3.8%)	9 (7.6%)	4 (1.8%)	
Hypertension	58 (16.8%)	58 (48.7%)	-	**<0.001**
Hyperlipidaemia	96 (27.8%)	96 (80.6%)	-	**<0.001**
Smoking				**<0.001**
Never smoked	309 (89.6%)	94 (79.0%)	215 (95.1%)	
Ex-smoker	24 (7.0%)	16 (13.4%)	8 (3.5%)	
Current smoker	12 (3.5%)	9 (7.6%)	3 (1.3%)	

**Variables are presented as absolute numbers (%), except for age and body mass index which will be presented as mean (standard deviation).

Bolded values indicate *p*-value of < 0.05 and it is taken as statistical significance.

### 3.2. Knowledge and attitude

#### 3.2.1. Hypertension

The majority of the population had heard of hypertension (89.6%), with a higher percentage of those with MFs being aware of hypertension (95.8%) compared to those without MFs (86.3%, *p* = 0.006). Of the study population, 68.7% agreed that hypertension is a lifelong disease. The large proportion of participants strongly agreed (52.8%) and agreed (38.3%) that hypertension will increase the risk of coronary artery disease and stroke; however, a substantial proportion of participants chose “neutral” (44.3%) when asked if hypertension will increase the risk of fatty liver disease.

Majority of participants felt that lifestyle changes (diet and exercise) were “very important” (74.2%) with regards to the control of blood pressure. Approximately two-thirds of participants found that being compliant to blood pressure medications was “very important” (67.2%) in managing hypertension. There was no difference in responses between those with and without MFs.

#### 3.2.2. Hyperlipidemia

Almost all participants had heard of hyperlipidemia (95.4%). While participants strongly agreed (33.0%) and agreed (43.2%) that hyperlipidemia will increase the risk of fatty liver disease, there was a higher proportion of those without MFs who strongly agreed (35.8%) compared to those with MFs (27.7%, *p* = 0.024). The majority of participants strongly agreed (58.0%) or agreed (33.0%) that hyperlipidemia will increase the risk of coronary artery disease and stroke, with no differences between the two groups (*p* = 0.099). Most participants felt that lifestyle changes were “very important” (72.8%) in controlling hyperlipidemia, with no difference in responses between those with and without MFs (*p* = 0.824).

#### 3.2.3. Diabetes Mellitus

Majority of the population had heard of diabetes mellitus (95.4%), with 78.6% of the population agreeing that it is a lifelong disease. The large proportion of participants strongly agreed (40.0%) and agreed (35.7%) that diabetes mellitus will increase the risk of coronary artery disease and stroke; however, more than a third of participants (36.2%) answered “neutral” when asked if diabetes mellitus will increase the risk of fatty liver disease. Almost all participants felt that lifestyle changes (84.9%) and adherence to prescribed medications (79.1%) were “very important” in controlling diabetes mellitus. There was no difference in responses between those with and without MFs.

#### 3.2.4. Non-alcoholic fatty liver disease

Most participants had heard of NAFLD (90.7%) and agreed that it can be reversed in its early stages (76.8%). A large proportion of participants agreed that fat in liver can cause serious health problems (90.4%) and liver failure (81.8%). There were no differences in responses between those with and without MFs. Majority of the participants either strongly agreed (45.8%) or agreed (35.9%) that excessive alcohol intake would increase the risk of NAFLD and agreed (42.3%) that NAFLD will increase the risk of coronary artery disease and stroke (37). Those without MFs were more likely to agree that family history of NAFLD (46.9% vs. 34.5% respectively, *p* = 0.030), smoking (36.3% vs. 26.9% respectively, *p* = 0.043), obesity (42.9% vs. 34.5% respectively, *p* = 0.024) and physical inactivity (49.1% vs. 32.6% respectively, *p* = 0.010) will increase the risk of NAFLD, compared to those with MFs.

Many of the participants felt that lifestyle factors were “very important” (72.8%) in treating NAFLD, with a larger proportion of those with MFs choosing “very important” (74.8%) compared to those without MFs (71.7%, *p* = 0.019). However, while most participants felt that compliance to medications for metabolic diseases was “very important” (53.9%) in the treatment NAFLD, a larger proportion of participants without MFs chose this option (56.6%) than those with MFs (48.7%, *p* = 0.034).

**Figure 1:**
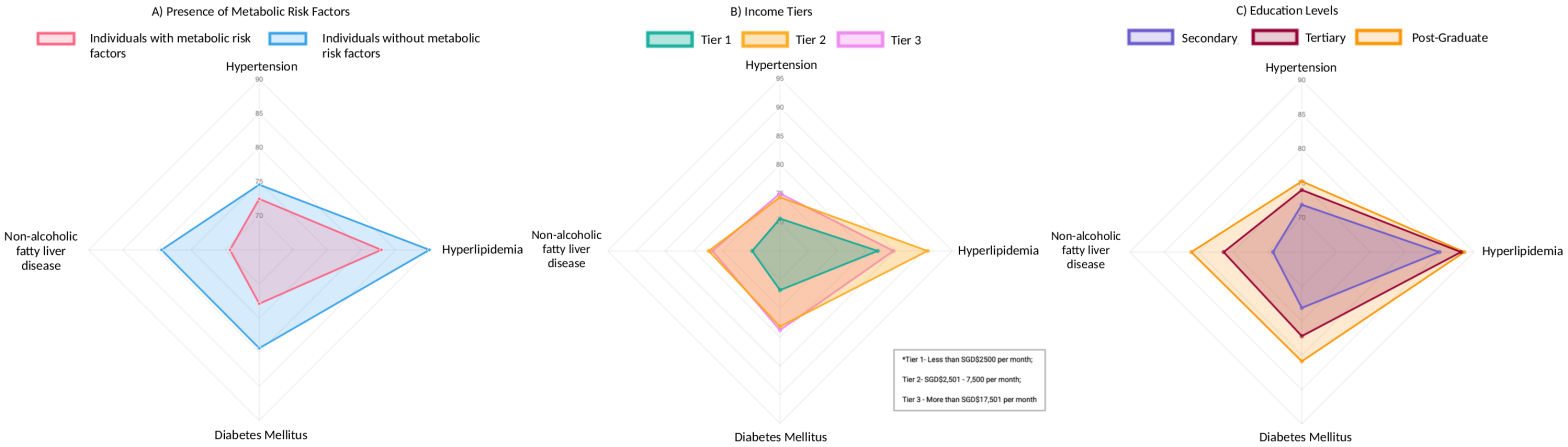
Radar figure of knowledge scores (presented as percentages) for each cardiometabolic disease stratified by **(A)** metabolic factors, **(B)** education level, and **(C)** income tiers.

Overall, participants had good knowledge of cardiometabolic diseases, answering 78.6% of all questions correctly. Participants demonstrated the best knowledge for hyperlipidemia (87.6% of questions answered correctly) and the biggest gaps in knowledge for hypertension (73.8% of questions answered correctly). Participants without MFs had higher knowledge scores on cardiometabolic diseases (80.8% of questions answered correctly) compared to those with MFs (74.4% of questions answered correctly) across all four cardiometabolic diseases. The biggest discrepancy in knowledge scores between participants with and without MFs was pertaining to NAFLD. There is no difference in knowledge scores across the income tiers apart from hyperlipidemia, with a trend toward higher knowledge scores with increasing levels of income. Knowledge scores improved as education level increased from secondary school education (74.8% questions answered correctly) to post-graduate (81.5% questions answered correctly) (Figure 1; Table 2).

**Table 2:** Attitude and knowledge with regards to cardiometabolic diseases.

Questions	Response	Overall	No metabolic risk factor	Metabolic risk factor	*p*-value
**Hypertension**					
*Knowledge*					
Have you heard of hypertension?	Yes	309 (89.6%)	195 (86.3%)	114 (95.8%)	**0.010**
	No	36 (10.4%)	31 (13.7%)	5 (4.2%)	
Do you think that high blood pressure is a lifelong disease?	Yes	237 (68.7%)	152 (67.3%)	85 (71.4%)	0.501
	No/Do not Know	108 (31.3%)	74 (32.7%)	34 (28.6%)	
High blood pressure is likely to increase my risk of fatty liver disease	Strongly agree	63 (18.3%)	44 (19.5%)	19 (16.0%)	**0.026**
	Agree	96 (27.8%)	74 (32.7%)	22 (18.5%)	
	Neutral	153 (44.3%)	90 (39.8%)	63 (52.9%)	
	Disagree	29 (8.4%)	16 (7.1%)	13 (10.9%)	
	Strongly disagree	4 (1.2%)	2 (0.9%)	2 (1.7%)	
High blood pressure is likely to increase my risk of coronary artery disease and stroke	Strongly agree	182 (52.8%)	124 (54.9%)	58 (48.7%)	0.373
	Agree	132 (38.3%)	85 (37.6%)	47 (39.5%)	
	Neutral	26 (7.5%)	14 (6.2%)	12 (10.1%)	
	Disagree	4 (1.2%)	3 (1.3%)	1 (0.8%)	
	Strongly disagree	1 (0.3%)	0	1 (0.8%)	
*Attitude*					
How important do you think lifestyle changes (diet and exercise) are with regards to control of your blood pressure?	Very important	256 (74.2%)	173 (76.5%)	83 (69.7%)	0.061
	Somewhat important	74 (21.4%)	48 (21.2%)	26 (21.8%)	
	Neutral	12 (3.5%)	4 (1.8%)	8 (6.7%)	
	Not very important	3 (0.9%)	1 (0.4%)	2 (1.7%)	
	Not important at all	0	0	0	
How important do you think it is for patients to take their prescribed medications to control blood pressure?	Very important	232 (67.2%)	158 (69.9%)	74 (62.2%)	0.289
	Somewhat important	91 (26.4%)	55 (24.3%)	36 (30.3%)	
	Neutral	20 (5.8%)	11 (4.9%)	9 (7.6%)	
	Not very important	2 (0.6%)	2 (0.9%)	0	
	Not important at all	0	0	0	
**Hyperlipidemia**					
*Knowledge*					
Have you ever heard of hyperlipidemia?	Yes	329 (95.4%)	215 (95.1%)	114 (95.8%)	0.992
	No	16 (4.6%)	11 (4.9%)	5 (4.2%)	
High cholesterol levels are likely to increase my risk of fatty liver disease	Strongly agree	114 (33.0%)	81 (35.8%)	33 (27.7%)	**0.024**
	Agree	149 (43.2%)	103 (45.6%)	46 (38.7%)	
	Neutral	73 (21.2%)	39 (17.3%)	34 (28.6%)	
	Disagree	6 (1.7%)	2 (0.9%)	4 (3.4%)	
	Strongly disagree	3 (0.9%)	1 (0.4%)	2 (1.7%)	
High cholesterol levels are likely to increase my risk of coronary artery disease and stroke	Strongly agree	200 (58.0%)	139 (61.5%)	61 (51.3%)	0.099
	agree	114 (33.0%)	72 (31.9%)	42 (35.3%)	
	Neutral	22 (6.4%)	9 (4.0%)	13 (10.9%)	
	Disagree	5 (1.4%)	3 (1.3%)	2 (1.7%)	
	Strongly Disagree	4 (1.2%)	3 (1.3%)	1 (0.8%)	
*Attitude*					
How important do you think lifestyle changes (diet and exercise) are to a patient’s cholesterol levels?	Very important	251 (72.8%)	169 (74.8%)	82 (68.9%)	0.824
	Somewhat important	80 (23.2%)	48 (21.2%)	32 (26.9%)	
	Neutral	6 (1.7%)	4 (1.8%)	2 (1.7%)	
	Not very important	3 (0.9%)	2 (0.9%)	1 (0.8%)	
	Not important at all	5 (1.4%)	3 (1.3%)	2 (1.7%)	
**Diabetes Mellitus**					
*Knowledge*					
Have you ever heard of diabetes?	Yes	329 (95.4%)	218 (96.5%)	111 (93.3%)	0.286
	No	16 (4.6%)	8 (3.5%)	8 (6.7%)	
Do you think that diabetes is a lifelong disease?	Yes	271 (78.6%)	175 (77.4%)	96 (80.7%)	0.576
	No	74 (21.4%)	51 (22.6%)	23 (19.3%)	
Diabetes is likely to increase my risk of fatty liver disease	Strongly agree	82 (23.8%)	60 (26.5%)	22 (18.5%)	0.099
	Agree	122 (35.4%)	84 (37.2%)	38 (31.9%)	
	Neutral	125 (36.2%)	75 (33.2%)	50 (42.0%)	
	Disagree	14 (4.1%)	6 (2.7%)	8 (6.7%)	
	Strongly disagree	2 (0.6%)	1 (0.4%)	1 (0.8%)	
Diabetes is likely to increase my risk of coronary artery disease and stroke	Strongly agree	138 (40.0%)	99 (43.8%)	39 (32.8%)	0.095
	Agree	123 (35.7%)	82 (36.3%)	41 (34.5%)	
	Neutral	61 (17.7%)	32 (14.2%)	29 (24.4%)	
	Disagree	18 (5.2%)	10 (4.4%)	8 (6.7%)	
	Strongly disagree	5 (1.4%)	3 (1.3%)	2 (1.7%)	
*Attitude*					
How important do you think lifestyle changes (diet and exercise) are to control your blood sugar levels?	Very important	293 (84.9%)	193 (85.4%)	100 (84.0%)	0.335
	Somewhat important	43 (12.5%)	29 (12.8%)	14 (11.8%)	
	Neutral	8 (2.3%)	3 (1.3%)	5 (4.2%)	
	Not very important	1 (0.3%)	1 (0.4%)	0	
	Not important at all	0	0	0	
How important is it for patients to take their prescribed diabetes medications regularly to control your blood sugar levels?	Very important	273 (79.1%)	181 (80.1%)	92 (77.3%)	0.471
	Somewhat important	59 (17.1%)	39 (17.3%)	20 (16.8%)	
	Neutral	10 (2.9%)	5 (2.2%)	5 (4.2%)	
	Not very important	3 (0.9%)	1 (0.4%)	2 (1.7%)	
	Not important at all	0	0	0	
**Non-alcoholic fatty liver disease**					
*Knowledge*					
Have you ever heard of fatty liver disease?	Yes	313 (90.7%)	210 (92.9%)	103 (86.6%)	0.081
	No	32 (9.3%)	16 (7.1%)	16 (13.4%)	
Do you think fatty liver disease can be reversible in its early stage?	Yes	265 (76.8%)	179 (79.2%)	86 (72.3%)	0.188
	No/do not know	80 (23.2%)	47 (20.8%)	33 (27.7%)	
Do you think fat in the liver can cause serious health problems?	Yes	312 (90.4%)	207 (91.6%)	105 (88.2%)	0.415
	No/do not know	33 (7.6%)	19 (8.4%)	14 (11.8%)	
Can fatty liver disease cause liver failure?	Yes	279 (81.1%)	185 (81.9%)	94 (79.7%)	0.617
	No/do not know	66 (18.9%)	41 (18.1%)	25 (21.0%)	
Family history of fatty liver disease is likely to increase the risk of fatty liver disease	Strongly agree	75 (21.7%)	53 (23.5%)	22 (18.5%)	**0.030**
	Agree	147 (42.6%)	106 (46.9%)	41 (34.5%)	
	Neutral	100 (29.0%)	55 (24.3%)	45 (37.8%)	
	Disagree	20 (5.8%)	10 (4.4%)	10 (8.4%)	
	Strongly disagree	3 (0.9%)	2 (0.9%)	1 (0.8%)	
Smoking is likely to increase the risk of fatty liver disease	Strongly agree	83 (24.1%)	59 (26.1%)	24 (20.2%)	**0.043**
	Agree	114 (33.0%)	82 (36.3%)	32 (26.9%)	
	Neutral	127 (36.8%)	76 (33.6%)	51 (42.9%)	
	Disagree	19 (5.5%)	8 (3.5%)	11 (9.2%)	
	Strongly disagree	2 (0.6%)	1 (0.4%)	1 (0.8%)	
Obesity is likely to increase the risk of fatty liver disease	Strongly agree	165 (47.8%)	111 (49.1%)	54 (45.4%)	**0.024**
	Agree	138 (40.0%)	97 (42.9%)	41 (34.5%)	
	Neutral	38 (11.0%)	16 (7.1%)	22 (18.5%)	
	Disagree	2 (0.6%)	1 (0.4%)	1 (0.8%)	
	Strongly disagree	2 (0.6%)	1 (0.4%)	1 (0.8%)	
Excessive stress is likely to increase the risk of fatty liver disease	Strongly agree	62 (18.0%)	39 (17.3%)	23 (19.3%)	0.164
	Agree	121 (35.1%)	89 (39.4%)	32 (26.9%)	
	Neutral	133 (38.6%)	82 (36.3%)	51 (42.8%)	
	Disagree	24 (7.0%)	14 (6.2%)	10 (8.4%)	
	Strongly disagree	5 (1.4%)	2 (0.9%)	3 (2.5%)	
Excessive alcohol intake is likely to increase the risk of fatty liver disease	Strongly agree	158 (45.8%)	106 (46.9%)	52 (43.7%)	0.083
	Agree	124 (35.9%)	87 (38.5%)	37 (31.1%)	
	Neutral	60 (17.4%)	32 (14.2%)	28 (23.5%)	
	Disagree	0	0	0	
	Strongly disagree	3 (0.9%)	1 (0.4%)	2 (1.7%)	
Physical inactivity is likely to increase the risk of fatty liver disease	Strongly agree	116 (33.6%)	75 (33.2%)	41 (34.5%)	**0.010**
	Agree	151 (43.8%)	111 (49.1%)	40 (32.6%)	
	Neutral	63 (18.2%)	30 (13.2%)	33 (27.7%)	
	Disagree	9 (2.6%)	6 (2.7%)	3 (2.5%)	
	Strongly Disagree	6 (1.7%)	4 (1.8%)	2 (1.7%)	
Fatty liver disease is likely to increase the risk of coronary artery disease and stroke	Strongly Agree	110 (31.9%)	77 (34.1%)	33 (27.7%)	0.279
	Agree	146 (42.3%)	99 (43.8%)	47 (39.5%)	
	Neutral	78 (22.6%)	43 (19.0%)	35 (29.4%)	
	Disagree	8 (2.3%)	5 (2.2%)	3 (2.5%)	
	Strongly Disagree	3 (0.9%)	2 (0.9%)	1 (0.8%)	
*Attitude*					
How important do you think lifestyle changes (diet and exercise) are in treating fatty liver disease?	Very important	251 (72.8%)	162 (71.7%)	89 (74.8%)	**0.019**
	Somewhat important	79 (22.9%)	59 (26.1%)	20 (16.8%)	
	Neutral	6 (1.7%)	2 (0.9%)	4 (3.4%)	
	Not very important	0	0	0	
	Not important at all	9 (2.6%)	3 (1.3%)	6 (5.0%)	
How important do you think taking medications regularly for metabolic diseases (such as diabetes and high cholesterol) is in treating fatty liver disease?	Very important	186 (53.9%)	128 (56.6%)	58 (48.7%)	**0.034**
	Somewhat important	108 (31.3%)	75 (33.2%)	33 (27.7%)	
	Neutral	45 (13.0%)	21 (9.3%)	24 (20.2%)	
	Not very important	4 (1.2%)	1 (0.4%)	3 (2.5%)	
	Not important at all	2 (0.6%)	1 (0.4%)	1 (0.8%)	

Bolded values indicate *p* < 0.05 and it is taken as statistical significance.

Some categories may not total 100% due to rounding.

### 3.3. Perceived risk

**Figure 2:**
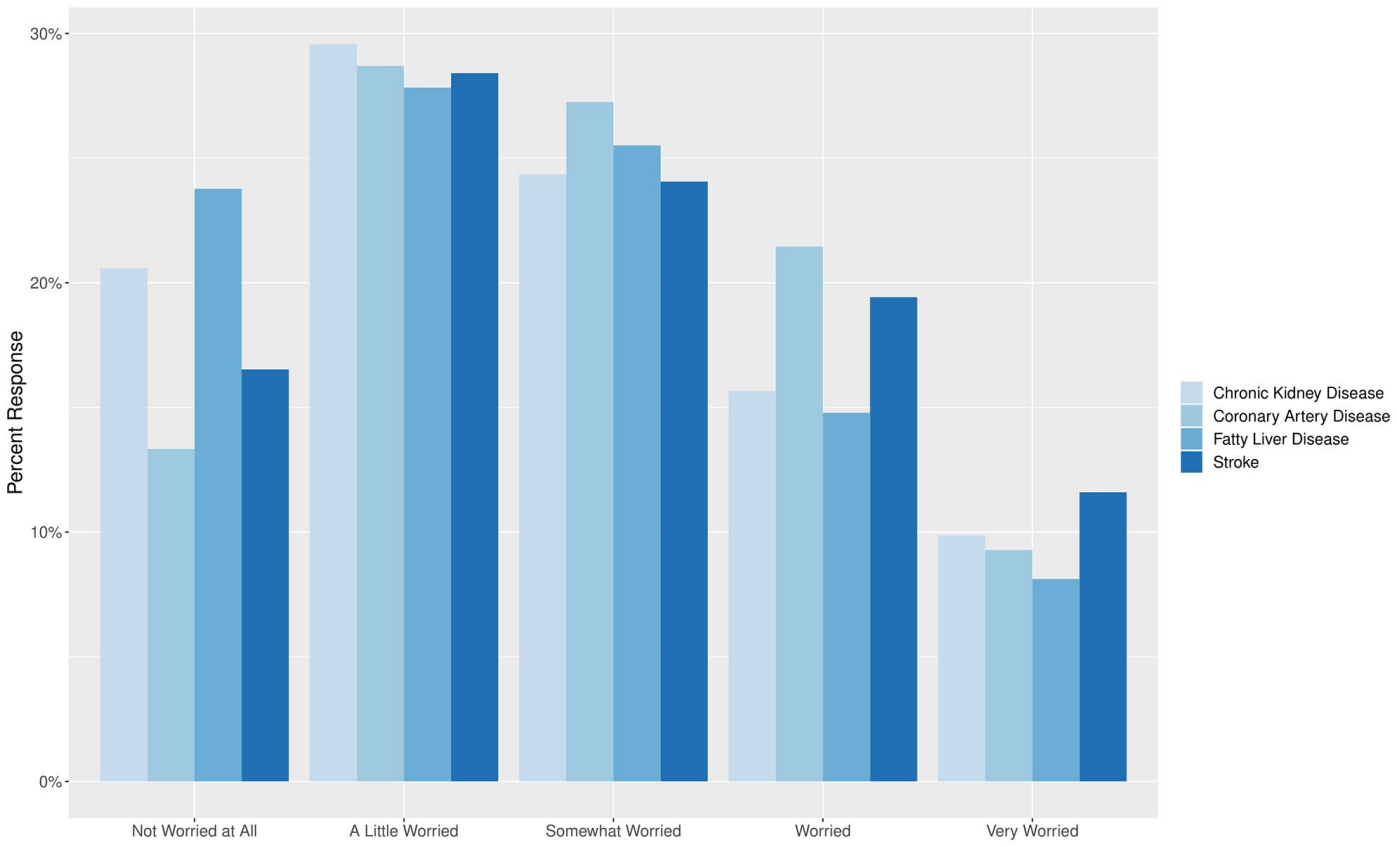
The level of concern for developing cardiometabolic diseases.

In the study cohort, the majority estimated their risk to be “low risk” (25.4%) or “neutral” (51.0%) for coronary artery disease, stroke, NAFLD, and chronic kidney disease. However, those with MFs were more likely to choose “very high risk” (4.4%) or “high risk” (25.9%) for all cardiometabolic complications compared to those without MFs (0.9% very high risk, 10.1% high risk). Similarly, majority of participants were only “somewhat worried” (25.3%) or “a little worried” (28.6%) about their risk of developing cardiometabolic diseases and complications. They were most worried about developing strokes (31.0% worried or very worried) and least concerned about incident NAFLD (22.9% worried or very worried). Those with MFs were generally more worried about developing coronary artery disease, stroke, NAFLD and chronic kidney disease compared to those without MFs (Figure 2; Table 3).

**Table 3:** Perceived risks and actions taken for cardiometabolic diseases.

Questions	Response	Overall (%)	No Metabolic Risk Factor	Metabolic Risk Factor	*p-*value
**Perceived risk**					
How do you estimate your risk of developing coronary artery disease (blockage of blood vessels of the heart)?	Very high risk	10 (2.9%)	1 (0.4%)	9 (7.6%)	**<0.001**
	High Risk	65 (18.8%)	29 (12.8%)	36 (30.3%)	
	Neutral	165 (47.8%)	115 (50.9%)	50 (42.0%)	
	Low Risk	86 (24.9%)	68 (30.1%)	18 (15.1%)	
	Very Low Risk	19 (5.5%)	13 (5.8%)	6 (5.0%)	
How do you estimate your risk of developing stroke?	Very High Risk	5 (1.4%)	2 (0.9%)	3 (2.5%)	**<0.001**
	High Risk	63 (18.3%)	22 (9.7%)	41 (34.5%)	
	Neutral	173 (50.1%)	121 (53.5%)	52 (43.7%)	
	Low Risk	83 (24.1%)	66 (29.2%)	17 (14.3%)	
	Very Low Risk	21 (6.1%)	15 (6.6%)	6 (5.0%)	
How do you estimate your risk of developing fatty liver disease (an abnormal accumulation of fats in the liver cells)?	Very High Risk	9 (2.6%)	4 (1.8%)	5 (4.2%)	**0.003**
	High Risk	52 (15.1%)	25 (11.1%)	27 (22.7%)	
	Neutral	174 (50.4%)	114 (50.4%)	60 (50.4%)	
	Low Risk	87 (25.2%)	69 (30.5%)	18 (15.1%)	
	Very low risk	23 (6.7%)	14 (6.2%)	9 (7.6%)	
How do you estimate your risk of developing chronic kidney disease?	Very high risk	5 (1.4%)	1 (0.4%)	4 (3.4%)	**<0.001**
	High risk	34 (9.9%)	15 (6.6%)	19 (16.0%)	
	Neutral	192 (55.7%)	121 (53.5%)	71 (60.0%)	
	Low risk	94 (27.3%)	76 (33.6%)	18 (15.1%)	
	Very low risk	20 (5.8%)	13 (5.8%)	7 (5.9%)	
How worried are you about developing coronary artery disease (blockage of blood vessels of the heart)?	Very worried	32 (9.3%)	13 (5.8%)	19 (16.0%)	**0.034**
	Worried	74 (21.4%)	50 (22.1%)	24 (20.2%)	
	Somewhat worried	94 (27.2%)	61 (27.0%)	33 (27.7%)	
	A little worried	99 (28.7%)	70 (31.0%)	29 (24.4%)	
	Not worried at all	46 (13.3%)	32 (14.2%)	14 (11.8%)	
How worried are you about getting a stroke?	Very Worried	40 (11.6%)	18 (8.0%)	22 (18.5%)	**0.023**
	Worried	67 (19.4%)	45 (19.9%)	22 (18.5%)	
	Somewhat worried	83 (24.1%)	51 (22.6%)	32 (26.9%)	
	A little worried	98 (28.4%)	70 (31.0%)	28 (23.5%)	
	Not	57 (16.5%)	42 (18.6%)	15 (12.6%)	
How worried are you about developing fatty liver disease?	Very Worried	28 (8.1%)	13 (5.8%)	15 (12.6%)	0.063
	Worried	51 (14.8%)	33 (14.6%)	18 (15.1%)	
	Somewhat Worried	88 (25.5%)	53 (23.5%)	35 (29.4%)	
	A Little Worried	96 (27.8%)	71 (31.4%)	25 (21.0%)	
	Not Worried at All	82 (23.8%)	56 (24.8%)	26 (21.8%)	
How worried are you about developing chronic kidney disease?	Very Worried	34 (9.9%)	18 (8.0%)	16 (13.4%)	0.152
	Worried	54 (15.7%)	32 (14.2%)	22 (18.5%)	
	Somewhat Worried	84 (24.3%)	52 (23.0%)	32 (26.9%)	
	A Little Worried	102 (29.6%)	73 (32.3%)	29 (24.4%)	
	Not Worried at All	71 (20.6%)	51 (22.6%)	20 (16.8%)	
**Actions taken**					
In a typical week, how many minutes of moderate intensity exercise do you perform? (I.e., you are working hard enough to raise your heart rate and break a sweat/you’ll be able to talk, but not be able to sing a song)	≤90 min^	235 (68.1%)	163 (72.1%)	72 (60.5%)	0.060
	91–149 min^	59 (1 7.1%)	36 (15.9%)	23 (19.3%)	
	≥150 min*	51 (14.8%)	27 (11.9%)	24 (20.2%)	
In a typical week, how many minutes of vigorous intensity exercise do you perform? (I.e., you are breathing hard and fast. You will not be able to say more than a few words without pausing for a breath)	≤30 min^	210 (60.9%)	133 (58.8%)	77 (64.7%)	0.390
	31–75 min^	100 (29.0%)	71 (31.4%)	29 (24.4%)	
	≥75 min*	35 (10.1%)	22 (9.7%)	13 (10.9%)	
I make a conscious effort to reduce the amount of fat in my diet	Strongly Agree	32 (9.3%)	17 (7.5%)	15 (12.6%)	0.081
	Agree	160 (46.4%)	102 (45.1%)	58 (48.7%)	
	Neutral	98 (28.4%)	63 (27.9%)	35 (29.4%)	
	Disagree	44 (12.8%)	34 (15.0%)	10 (8.4%)	
	Strongly disagree	11 (3.2%)	10 (4.4%)	1 (0.8%)	
I make a conscious effort to not exceed the recommended caloric intake in my diet (Aim 2,200 calories for Males and 1800 calories for Females)	Strongly agree	19 (5.5%)	13 (5.8%)	6 (5.0%)	0.238
	Agree	90 (26.1%)	53 (23.5%)	37 (31.1%)	
	Neutral	123 (35.7%)	77 (34.1%)	46 (38.7%)	
	Disagree	88 (25.5%)	64 (28.3%)	24 (20.2%)	
	Strongly disagree	25 (7.2%)	19 (8.4%)	6 (5.0%)	
I make a conscious effort to reduce the amount of sugar in my diet (<10 teaspoon of sugar a day)	Strongly agree	92 (26.7%)	52 (23.0%)	40 (33.6%)	0.103
	Agree	150 (43.5%)	97 (42.9%)	53 (44.5%)	
	Neutral	51 (14.8%)	37 (16.4%)	14 (11.8%)	
	Disagree	38 (11.0%)	30 (13.3%)	8 (6.7%)	
	Strongly disagree	14 (4.1%)	10 (4.4%)	4 (3.4%)	
I make a conscious effort to reduce the salt intake in my diet (<2 g of salt a day/do not add additional salt to meals)	Strongly agree	36 (10.4%)	22 (9.7%)	14 (11.8%)	0.067
	Agree	131 (38.0%)	77 (34.1%)	54 (45.4%)	
	Neutral	104 (30.1%)	69 (30.5%)	35 (29.4%)	
	Disagree	56 (16.2%)	45 (19.9%)	11 (9.2%)	
	Strongly disagree	18 (5.2%)	13 (5.8%)	5 (4.2%)	
I make a conscious effort to reduce red meat and processed meat consumption (sausage and ham etc.)	Strongly agree	41 (11.9%)	29 (12.8%)	12 (10.1%)	**0.045**
	Agree	132 (38.3%)	74 (32.7%)	58 (48.7%)	
	Neutral	87 (25.2%)	59 (26.1%)	28 (23.5%)	
	disagree	68 (19.7%)	52 (23.0%)	16 (13.4%)	
	Strongly disagree	17 (4.9%)	12 (5.3%)	5 (4.2%)	
I make a conscious effort to eat at least 2 servings of fruits and 2 servings of vegetables a day (one serving is one cup)	Strongly agree	36 (10.4%)	18 (8.0%)	18 (15.1%)	**0.009**
	Agree	145 (42.0%)	95 (42.0%)	50 (42.0%)	
	Neutral	93 (27.0%)	56 (24.8%)	37 (31.1%)	
	Disagree	62 (18.0%)	48 (21.2%)	14 (11.8%)	
	Strongly disagree	9 (2.6%)	9 (4.0%)	0 (0.0%)	

Bolded values indicate *p* < 0.05 and it is taken as statistical significance.

Some categories may not total 100% due to rounding.

*Indicates the correct answer(s) based on guidelines, ^ Indicates the wrong answers.

Multivariable linear regression demonstrated that the presence of MFs (adjusted β: 2.752, 95%CI: 0.772 to 4.733, *p* = 0.007) was significantly associated with increased perceived risk after adjusting for age, sex, race, education, and marital status. Higher knowledge scores of cardiometabolic diseases (adjusted β: 0.418, 95%CI: 0.236 to 0.600, *p* < 0.001) were also associated with higher perceived risk, after adjusting for important confounders (Table 4).

**Table 4:** Independent predictors of the individual’s perceived risk of cardiometabolic diseases.

Risk factor	β	95% CI	*p*-value
Age	−0.007	−0.076 to 0.063	0.852
Sex (Male)	−0.104	−1.667 to 1.459	0.896
*Ethnicity*			
*Chinese*	Reference		
*Malay*	2.391	−1.065 to 5.848	0.174
*Indian*	1.574	−3.154 to 6.302	0.513
*Others*	−2.981	−7.755 to 1.794	0.220
*Marital status*			
*Never married*	Reference		
*Married*	0.332	−1.413 to 2.077	0.709
*Divorced*	1.825	−2.628 to 6.278	0.421
*Widowed*	1.908	−4.076 to 7.892	0.531
*Education status*			
*Secondary Education*	Reference		
*Tertiary Education*	0.715	−1.295 to 2.724	0.485
*Post-Graduate*	0.465	−1.848 to 2.779	0.693
Metabolic risk factors	2.752	0.772 to 4.733	**0.007**
Knowledge of diseases	0.418	0.236 to 0.600	**<0.001**

Bolded values indicate *p*<0.05 and it is taken as statistical significance.

Secondary Education: Secondary school, Junior college, Polytechnic and ITE; Tertiary Education: University Bachelor’s Degree or equivalent; Post-Graduate: University Master’s Degree/PhD.

### 3.4. Actions taken

**Figure 3:**
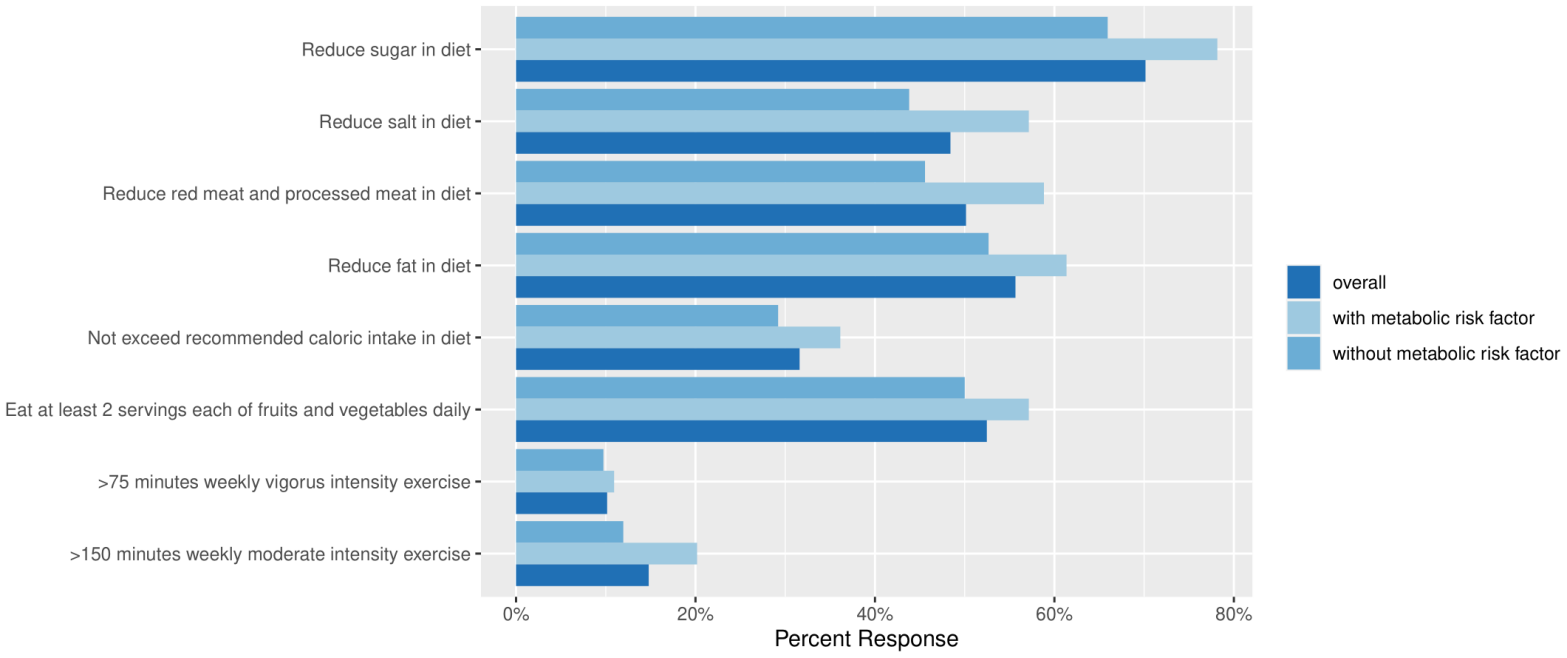
The percentage of study population undertaking actions to improve metabolic health, stratified by the presence of metabolic risk factors.

Only a minority adhered to the recommended guidelines of more than 150 min of moderate intensity exercise (14.8%) and more than 75 min of vigorous-intensity exercise per week (10.1%). Most participants made a conscious effort in cutting down sugar (70.1%), dietary fat (55.7%), red and processed meat (50.1%) in their diet. However, less than half of participants made a conscious effort to reduce salt in their diet (48.4%) or to limit their daily caloric intake (31.6%). Only 35.1% of participants followed the healthy plate recommendations for at least one meal daily. Nevertheless, those with MFs were more likely to agree or strongly agree with making a conscious effort in reducing red and processed meat consumption (*p* = 0.009), as well as having at least 2 servings of fruits and vegetables a day (*p* = 0.045), compared to those without MFs (Figure 3, Table 3).

## 4. Discussion

Our findings are concordant with current literature, showing heterogeneity in the knowledge across various cardiometabolic diseases and subgroups of the population (38). In recent years, cardiometabolic diseases have been on the rise across the globe (39), especially in Asia, attributed to globalization, socioeconomic changes, technological advancements, and sedentary living (5, 40–42). The study extends the current literature by providing a comprehensive overview of the associations between knowledge, attitudes, perceived risk, and actions taken toward cardiometabolic health in a multi-ethnic Singaporean population. There are several pertinent findings: (1) knowledge on cardiometabolic diseases and the presence of MFs are closely linked to an individual’s perception of disease; (2) The largest knowledge gap among all the cardiometabolic diseases pertains to hypertension. However, when comparing to individuals without MFs, those with MFs had lower knowledge scores on all cardiometabolic domains with the largest discrepancy demonstrated in knowledge scores related to NAFLD; (3) Despite the increased perceived risk in those with MFs, this translated to only a few increased self-reported actions taken to address metabolic health, compared to those without MFs – namely red meat and processed food reduction, and adequate fruits/vegetables intake. The lowest adherence to preventive recommendations pertained to those related with physical activity. The study highlights a vulnerable group of individuals living with cardiometabolic disease, with lower levels of knowledge scores and self-reported action plans, that appear discordant to the self-perceived risk of cardiometabolic complications.

The study highlights that the increased knowledge on cardiometabolic diseases is closely associated with an increase in the individual’s perceived risk of cardiometabolic disease. We postulate that this observation may be attributed to public education and awareness of cardiometabolic diseases, thus equipping individuals to comprehend the modifiable lifestyle factors that can mitigate the risk of metabolic diseases. Evidence has shown that education remains the key component in primary prevention (24, 43). However, for this to be effective, the “knowledge-to-action gap” where the knowledge of individuals fails to translate to tangible behavioral change needs to be concurrently addressed (44). Based on previous studies, knowledge of a condition must be coupled with knowledge of the treatment and the reasons for treatment compliance (45–47). With a strong baseline understanding of the condition, channeling resources into helping patients understand how daily efforts to improve their diet or increase physical activity can positively impact their condition (48–50). This may be effective in bridging the gap between knowledge of a condition and actions taken. Coupled with improving health literacy in our modern-day society, there is an increasing role for active public involvement and empowering individuals to take charge of their own health. Healthier SG (51) is a national program in Singapore that focuses on empowering the public in understanding and taking initiative toward adopting preventive measures to sustain health within the community and to manage their chronic conditions proactively.

The presence of MFs can also alter the perception and knowledge of cardiometabolic diseases. Those with MFs were more likely to have higher perceived risk of metabolic disease complications. This is important as self-perceived susceptibility to cardiometabolic diseases can serve as motivation toward taking early preventive measures, which in turn modifies the course of cardiometabolic risks and their complications (52). However, the study highlights several gaps that need to be addressed. Those with MFs had lower knowledge scores on all cardiometabolic domains when compared to their counterparts without MFs. Although it is plausible that the higher baseline knowledge of cardiometabolic diseases has equipped individuals to take preemptive measures early to mitigate the risk of cardiometabolic diseases (53), this still raises concerns over the discrepancy in the knowledge gap in those living with cardiometabolic diseases (54, 55). Despite the increased perceived susceptibility of those with MFs, it is concerning that this did not appear to translate into self-reported actions taken to improve metabolic health. This may be due to the lack of knowledge on the cardiometabolic diseases, lack of self-efficacy or motivation as suggested by previous studies (52). Previous studies have also shown that an individual’s health literacy is key in the self-management of cardiometabolic diseases (56, 57)^.^ We demonstrated that the knowledge scores increased across all cardiometabolic diseases with higher levels of education, but not with higher income. This may be attributed to higher levels of health literacy (58), with education on cardiometabolic diseases playing a significant role in disease prevention. While social economic status may play a part, its role is less defined. Future studies could explore the discrepancies and the barriers that individuals with MFs may experience in trying to mitigate their cardiometabolic risks.

There were several disease-specific knowledge gaps that should be addressed in public education efforts. Knowledge on hyperlipidemia received the highest scores, while the lowest scores were observed for hypertension (59). This is concerning because hypertension is the commonest chronic condition in Singapore. Previous studies have suggested collaborative health education between patients and clinicians as a cornerstone in improving self-management of hypertension (60). Interestingly, participants shared the least concern toward the cardiometabolic complications of NAFLD (61, 62), and we postulate that the reasons for this observation may be multifactorial (63). The greatest cardiometabolic concern for participants was the chance of getting a stroke, with the least concern shown for NAFLD. We postulate that the lack of concern for NAFLD is a result of the following factors; first, there is an underdiagnosis of NAFLD by physicians across the globe (64–67), possibly contributed by the knowledge gap in the identification and diagnosis of NAFLD, as well as the limited treatment options in NAFLD (63, 68, 69). Second, less media portrayal and patient education on NAFLD, relative to that of stroke and coronary artery disease, may have been contributing factors (70, 71). Consequently, the above factors may translate to a lower level of awareness of NAFLD, its severity and consequences, among the general population.

Within the Asian community, studies have shown that the general population tend to lead sedentary lifestyles, spending a significant amount of time sitting down at work, while commuting, and during leisure activities (72, 73). Although most study participants tried to improve their diets by reducing their sugar, red meat, and processed foods intake, only a minority made conscious efforts to increase physical activity, with less than a sixth of participants achieving the Health Promotion Board recommendations of 150 min of moderate-intensity or 75 min of vigorous-intensity physical activity per week (74). This showcases the need for greater public health messaging to incentivize individuals to adopt a more active lifestyle. One such example is the National Steps Challenge that gives participants instant rewards for clocking in steps and doing moderate-vigorous physical activities (75). While these national initiatives are promising forays in encouraging the adoption of physical activity nationwide (76), they share the common pitfalls of other similar mobile health initiatives. Leveraging on novelty and financial incentives, participants were only actively engaged in the first third of the National Steps Challenge programme (77). This highlights the need for other social, cultural and environmental factors (78) to be concurrently addressed for the benefit of lifestyle interventions to be sustainable. At present, the mindset within the population may not be completely receptive toward preventive measures, with the National Population Health Survey 2021 reporting that a substantial proportion of the public expressing that it is not necessary to go for health screenings as they are already healthy (5).

Moving forward, the findings from this study can be used to tailor appropriate healthcare policies, especially towards the Singaporean population. In the 21st century, with an increasingly obesogenic environment, coupled with poor nutrition and sedentarism (54, 79, 80), the burden of cardiometabolic diseases is expected to rise further in Asia (81, 82). It is therefore paramount that early action is taken to mitigate this public health burden. Our study emphasizes the importance of targeting the public’s knowledge on cardiometabolic diseases, which is closely associated with the perception of disease and risk. This can serve as catalysts for necessary changes to the attitudes and preventive actions taken to sustain metabolic health (83). Furthermore, the study identifies the vulnerable group of individuals living with metabolic disease, with lower levels of knowledge and action plans that is discordant to their increased perceived risk. Our findings highlight an urgent need to modify policy development in implementing more targeted preventive strategies with an emphasis on the population with metabolic diseases. Interventions should target the root causes of cardiometabolic diseases by tackling issues with knowledge, attitudes and perception, and lifestyle modifications (53, 84). As these metabolic diseases do not exist in silos, prioritizing upstream solutions can help mitigate the overall metabolic milieu of the individual, with the potential benefit of reducing downstream healthcare demands and expenditure.

### 4.1. Study limitations

This study is the first to explore knowledge, attitudes, and perceived risk of cardiometabolic diseases and the action taken to mitigate cardiometabolic risks in a multi-ethnic Singaporean population. However, the study has its limitations. First, as the study used convenience sampling, results may not be generalizable to the entire population, and hence the study results should be interpreted with caution. Although our preliminary results are hypothesis-generating, the findings shed light on the understudied perceptions and attitudes of the general public toward the rising metabolic epidemic. This provides an important platform for future systematic probabilistic sampling with adequately powered sample size calculations to validate the current findings. Second, due to the voluntary nature of the survey, individuals who participate in the survey may tend to have differential concerns of their health, thus introducing self-selection bias to the included study population. This selection bias may lead to an overestimation of the awareness of cardiometabolic disease in the Singapore population. Third, while the questionnaire we used was validated in other published studies, they have not been validated in a Singaporean population. Fourth, the survey was distributed in English, hence there may be a risk in response errors especially among those with limited proficiency in English.

## 5. Conclusion

In the silent metabolic disease epidemic, there appears to be disparate levels of knowledge, attitude, perceived risks, and actions taken toward metabolic health in the study population living in Singapore. The preliminary study findings highlighted a vulnerable subpopulation of individuals living with metabolic risk factors, with high perceived risks, and discordant levels of knowledge and preventive actions taken. Future powered studies with systematic probabilistic sampling will be the next important step to validate the study findings. Nevertheless, our preliminary findings suggest that consolidated efforts should be channeled into addressing the knowledge-to-action gap, with the unified goal of sustaining optimal metabolic health.

## Data availability statement

The raw data supporting the conclusions of this article will be made available by the authors, without undue reservation.

## Ethics statement

The studies involving human participants were reviewed and approved by National Healthcare Group Domain Specific Review Board. Written informed consent for participation was not required for this study in accordance with the national legislation and the institutional requirements.

## Author contributions

VA and RG conceptualized, acquired, and analyzed data. BN, SK, and NC designed the work and interpreted the data. JL, NN, JC, YL, YC, BC, GK, BT, ZL, CK, LG, PL, PC, MD, MC, RF, and MM drafted the work and substantially revised it. All authors read and approved the final manuscript.

## Conflict of interest

The authors declare that the research was conducted in the absence of any commercial or financial relationships that could be construed as a potential conflict of interest.

## Publisher’s note

All claims expressed in this article are solely those of the authors and do not necessarily represent those of their affiliated organizations, or those of the publisher, the editors and the reviewers. Any product that may be evaluated in this article, or claim that may be made by its manufacturer, is not guaranteed or endorsed by the publisher.

## Abbreviations

MF: – Metabolic risk factor
NAFLD: – Non-alcoholic fatty liver disease
